# Long noncoding RNA Pvt1 regulates the immunosuppression activity of granulocytic myeloid-derived suppressor cells in tumor-bearing mice

**DOI:** 10.1186/s12943-019-0978-2

**Published:** 2019-03-30

**Authors:** Yu Zheng, Xinyu Tian, Tingting Wang, Xueli Xia, Fenghua Cao, Jie Tian, Ping Xu, Jie Ma, Huaxi Xu, Shengjun Wang

**Affiliations:** 1 Department of Laboratory Medicine, the Affiliated People’s Hospital, Jiangsu University, Zhenjiang, 212013 China; 2 Department of Immunology, Jiangsu Key Laboratory of Laboratory Medicine, School of Medicine, Jiangsu University, Zhenjiang, 212013 China; 3 Department of Laboratory Medicine, the Affiliated Wuxi People’s Hospital of Nanjing Medical University, Wuxi Children’s Hospital, Wuxi, China; 4 Department of Laboratory Medicine, The Fifth People’s Hospital of Suzhou, Suzhou, China; 5 Department of Laboratory Medicine, Zhenjiang Hospital of Chinese Traditional and Western Medicine, Zhenjiang, China

**Keywords:** Myeloid-derived suppressor cells, Long noncoding RNA, Pvt1, Immunosuppression

## Abstract

**Background:**

Myeloid-derived suppressor cells (MDSCs) participate in tumor-elicited immunosuppression by dramatically blocking T-cell-induced antitumor responses, thereby influencing the effectiveness of cancer immunotherapies. Treatments that alter the differentiation and function of MDSCs can partially restore antitumor immune responses. The long noncoding RNA plasmacytoma variant translocation 1 (lncRNA Pvt1) is a potential oncogene in a variety of cancer types. However, whether lncRNA Pvt1 is involved in the regulation of MDSCs has not been thoroughly elucidated to date.

**Methods:**

MDSCs or granulocytic MDSCs (G-MDSCs) were isolated by microbeads and flow cytometry. Bone marrow derived G-MDSCs were induced by IL-6 and GM-CSF. The expression of lncRNA Pvt1 was measured by qRT-PCR. Specific siRNA was used to knockdown the expression of lncRNA Pvt1 in G-MDSCs.

**Results:**

In this study, we found that knockdown of lncRNA Pvt1 significantly inhibited the immunosuppressive function of G-MDSCs in vitro. Additionally, lncRNA Pvt1 knockdown reduced the ability of G-MDSCs to delay tumor progression in tumor-bearing mice in vivo. Notably, lncRNA Pvt1 was upregulated by HIF-1α under hypoxia in G-MDSCs.

**Conclusions:**

Taken together, our results demonstrate a critical role for lncRNA Pvt1 in regulating the immunosuppression activity of G-MDSCs, and lncRNA Pvt1 might thus be a potential antitumor immunotherapy target.

**Electronic supplementary material:**

The online version of this article (10.1186/s12943-019-0978-2) contains supplementary material, which is available to authorized users.

## Background

Myeloid-derived suppressor cells (MSDCs) represent a heterogeneous population of immature myeloid cells (IMCs) and comprise myeloid progenitors and precursors of granulocytes, macrophages and dendritic cells (DCs), which are a type of immune-suppressive cell that suppresses T cell function [1–3]. In healthy individuals, IMCs quickly differentiate into mature granulocytes, macrophages or DCs after being generated in the bone marrow. However, under pathological conditions, such as cancer, infection, inflammation or autoimmune conditions, IMCs can be blocked from differentiation into mature myeloid cells, resulting in expansion of MDSCs [4–6]. In mice, MDSCs coexpress two myeloid cell lineage differentiation antigens, CD11b and Gr-1. Murine MDSCs can be further divided into two different subsets according to their morphology and phenotype: CD11b^+^Ly6G^+^Ly6C^low^ granulocytic MDSCs (G-MDSCs) and CD11b^+^Ly6G^−^Ly6C^high^ monocytic MDSCs (M-MDSCs) [7, 8]. G-MDSCs, which represent the majority of the MDSC population, comprise nearly 70–80% of all MDSCs in tumor-bearing mice and cancer patients [9–11]. It is widely known that G-MDSCs suppress T-cell-induced antitumor responses primarily through high levels of arginase 1 (Arg1) expression and reactive oxygen species (ROS) production, whereas M-MDSCs express inducible NO synthase (iNOS) in addition to a high level of Arg1 [7, 9, 12]. Therefore, compounds that reduce the levels of Arg1, ROS or iNOS in MDSCs, which can restore T-cell-induced antitumor responses, are considered to be potential antitumor immunotherapeutic agents [13–15].

Long noncoding RNAs (lncRNAs) have more than 200 nucleotides and are defined as a class of transcripts that do not have protein encoding information [16]. In recent years, according to the localization of lncRNAs relative to protein-coding target mRNAs, lncRNAs have been classified as intronic lncRNAs, antisense lncRNAs, long intergenic ncRNAs (lincRNAs), enhancer RNAs (eRNAs), or transcribed pseudogene lncRNAs [17]. It has become increasingly clear that lncRNAs participate in regulation of the function and development of myeloid cells and immune cells [18–22]. However, the molecular mechanism of lncRNAs in MDSC differentiation and function has not been thoroughly elucidated to date.

The mouse plasmacytoma variant translocation (Pvt1) gene has been identified as a candidate oncogene located at chromosome 15 and encodes a lincRNA homologous to that encoded by the human PVT1 gene, which maps to chromosome 8 [23–25]. In humans, increased copy number and overexpression of lncRNA PVT1 are tightly associated with a variety of cancer types, including hepatocellular carcinoma, gastric cancer, esophageal cancer, cervical cancer, bladder cancer and acute myeloid leukemia [26–31]. Moreover, lncRNA PVT1 shares a recognized cancer risk locus with the nearby, well-known MYC oncogene [23]. MYC is located on chromosome 8 in humans and has an equivalent in mice (on chromosome 15), and MYC expression is significantly increased in cancer. Coamplification of MYC and lncRNA PVT1 has been found in a variety of human and animal tumors in the past several decades. Moreover, the copy number of lncRNA PVT1 was found to be coincreased in more than 98% of tumors exhibiting increased MYC expression [32–34]. Although the mechanism underlying lncRNA Pvt1 activity in cancer cells has been elaborated in detail, how lncRNA Pvt1 regulates MDSC function and differentiation has not been elucidated to date.

Inhibition of MDSC function is a feasible approach to restore T-cell-induced antitumor immune responses [1, 14]. Our data provide the first evidence that lncRNA Pvt1 plays an important role in regulating the immunosuppressive capacity of G-MDSCs. LncRNA Pvt1 knockdown decreased the suppression of G-MDSCs and partially restored antitumor T-cell responses. Hypoxia-inducible factor (HIF)-1α upregulated lncRNA Pvt1 expression in G-MDSCs under hypoxia. These findings indicate that lncRNA Pvt1 may be a potential therapeutic target for regulating the suppressive function of G-MDSCs.

## Methods

### Cell line, mice and tumor models

Lewis lung carcinoma (LLC) cells were obtained from the American Type Culture Collection. C57BL/6 mice (6–8 weeks, 18–22 g, male) were purchased from the Animal Research Center of Jiangsu University (Zhenjiang, China) and housed under specific-pathogen-free conditions. LLC cells (2 × 10^6^/mouse) were implanted in mice via s.c. injection to construct tumor models. All experimental protocols were approved by the Committee on the Use of Live Animals in Research and Teaching of Jiangsu University.

### Tissue and cell preparation

Spleen, tibia, femur and tumor tissues were harvested when mice were sacrificed. Spleen cells were lysed with ACK buffer. Bone marrow cells were immediately flushed from tibia and femur and then lysed with ACK buffer. Tumor tissues were removed, cut into small pieces (1–2 mm^3^), and digested in RPMI 1640 (Gibco, Carlsbad, CA) medium supplemented with 5% fetal calf serum (FBS), 0.5 mg/ml collagenase type V, 0.2 mg/ml hyaluronidase and 0.015 mg/ml DNase I (Sigma-Aldrich, St. Louis, MO) for 2 h at 37 °C. Single-cell suspensions derived from removed organs were obtained using a 70-μm cell strainer.

### Isolation of G-MDSCs and CD4^+^T cells

Murine G-MDSCs were isolated from the bone marrow, spleen and tumor tissues of LCC tumor-bearing mice using a mouse MDSC isolation kit (Miltenyi Biotec, Auburn, CA). To improve the purity of G-MDSCs harvested from tumor tissues, Ly6G-enriched G-MDSCs were subsequently isolated using flow cytometry (FCM). In addition, murine CD4^+^ T cells were isolated from spleens of wild-type (WT) C57BL/6 mice using monoclonal anti-mouse CD4 antibodies conjugated to MicroBeads (Miltenyi Biotec, Auburn, CA). The purity of G-MDSCs and CD4^+^ T cells obtained from the isolated cells was confirmed by FCM.

### Flow cytometry

Single-cell suspensions were stained with relevant fluorochrome-conjugated mAbs: anti-mouse CD3, CD4, and CD8 antibodies from eBioscience (San Diego, CA) and anti-mouse CD11b, Gr-1, Ly6G and Ly6C antibodies from Biolegend (San Diego, CA). For detection of T helper 1 (Th1) cells and cytotoxic T lymphocytes (CTLs), single-cell suspensions from spleens, draining lymph nodes and tumor tissues were stimulated with 50 ng/mL PMA (Sigma-Aldrich, St. Louis, MO), 2 μg/mL ionomycin and 1 μg/mL monensin (eBioscience, San Diego, CA). After 5 h, the cells were stained with anti-CD3, anti-CD4, anti-CD8 mAbs; fixed; permeabilized; and stained with an anti-IFN-γ mAb according to instructions provided in an Intracellular Staining Kit (eBioscience, San Diego, CA). Flow cytometry was performed using FACSCalibur Flow Cytometer (Becton Dickinson).

### RNA isolation and quantitative real-time PCR (qRT-PCR)

Total RNA was extracted from cells using TRIzol (Invitrogen, Carlsbad, CA). Next, cDNA was synthesized with a Prime Script RT Reagent Kit (Takara, Osaka, Japan) according to the manufacturer’s instructions. Gene transcripts were quantified via real-time quantitative PCR performed with SYBR Premix Ex Taq (Tli RNaseH Plus) (Takara, Osaka, Japan). The sequences for the primers used were as follows: Pvt1, 5′-ATCCACCCTCTTGTCTGATTTTCT-3′ (forward) and 5′-AATCCACAGGGTTCAGGAAGTC-3′ (reverse); c-myc, 5′-AGCGACTCTGAAGAGCAAG-3′ (forward) and 5′-ATGGAGATGAGCCCGACTC-3′ (reverse); β-actin, 5′-TGGAATCCTGTGGCATCCATGAAAC-3′ (forward) and.

5′-TAAAACGCAGCTCAGTAACAGTCCG-3′ (reverse). Relative quantification of mRNA expression was calculated using the comparative threshold cycle (Ct) method.

### Western blotting

Protein extracted from cells was lysed in radioimmunoprecipitation (RIPA) buffer, and cell debris was removed by centrifugation. The extracts were quantified using a protein assay and then boiled in SDS gel-loading buffer containing 10% β-mercaptoethanol. Proteins were separated in 10% sodium dodecyl sulfate-polyacrylamide gel electrophoresis (SDS–PAGE) gels and then transferred onto immobilon PVDF membranes (Bio-Rad, Hercules, CA), which were probed with rabbit mAbs against mouse β-actin (CST, Danvers, MA), and c-myc and HIF-1α (Wanleibio, Co., Shenyang, China), and then incubated with HRP-conjugated goat anti-rabbit IgG antibody (Abcam, Cambridge, UK) followed by chemiluminescence detection (Champion Chemical, Whittier, CA).

### Transfection

G-MDSCs were plated in 48-well plates or 24-well plates (Costar, Corning, NY) with RPMI 1640 medium supplemented with 10% fetal calf serum and then transfected with 50 nM Pvt1 siRNA or the negative control (Ribobio Co., Guangzhou, China) using Entranster-R (Engreen Biosystem Co., Beijing, China) according to the manufacturer’s instructions. Six hours after transfection, 1 ng/mL GM-CSF was supplemented in culture system.

### Detection of arginase activity and ROS level

Arginase activity was detected using a quantitative colorimetric assay by employing a QuantiChrom Arginase Assay kit (BioAssay systems, Hayward, CA). Arginase activity was calculated according to the kit manufacturer’s instructions.

ROS produced by G-MDSCs was measured using the oxidation-sensitive dye 2′, 7′-dichlorofluorescin diacetate (Invitrogen, Carlsbad, CA). Cells were simultaneously cultured with 2.5 μM 2,7-dichlorofluorescin diacetate and 30 ng/mL PMA in PBS for 30 min. Then, ROS produced by G-MDSCs were detected by flow cytometry.

### Generation of BM-derived G-MDSCs

Tibia and femur from wild-type C57BL/6 mice were removed, and bone marrow cells were flushed from the bones. Erythrocytes were lysed with ACK for 5 min. To obtain BM-derived G-MDSCs, 1 × 10^^6^ bone marrow cells were plated in 24-well plates (Costar, Corning, NY) in 1 mL RPMI 1640 (Gibco, Carlsbad, CA) complete medium consisting of 10% fetal calf serum (FBS), 100 U/mL penicillin, 100 μg/ml streptomycin, 20 ng/mL IL-6 and 20 ng/mL GM-CSF (Peprotech, Rocky Hill, USA). After 3 days, the cells were collected and isolated using an MDSC Isolation Kit (Miltenyi Biotec, Auburn, CA) to acquire CD11b^+^ Ly6G^+^ G-MDSCs.

### Assessment of G-MDSC suppressive function

G-MDSCs were transfected with Pvt1 siRNA or the negative control. Responder cells (splenic CD4^+^ T cells) were cocultured with transfected G-MDSCs in U-bottomed 96-well plates (Costar, Corning, NY) in the presence of 10 μg/mL anti-CD3 mAb and 5 μg/mL anti-CD28 mAb (Biolegend, San Diego, CA) for 72 h and then pulsed with [^3^H]-thymidine (Pharmacia Biotech, Stockholm, Sweden, 1 μCi/well) for the last 16 h of culture. The capacity of G-MDSCs to suppress T cells was calculated according to the cpm value.

To detect the suppression function of G-MDSCs induced from bone marrow cells using IL-6 and GM-CSF treatment. 1 × 10^7^/mL splenic CD4^+^ T cells were stained with the fluorescent dye CFSE (5 μM, Invitrogen) at 37 °C for 10 min and keep protected from light. 5 times the original staining volume of RPMI 1640 (Gibco, Carlsbad, CA) complete medium consisting of 10% fetal calf serum were added, pellet cells were washed twice with RPMI 1640 medium. CFSE labeled CD4^+^ T cells were cocultured with BM-derived G-MDSCs in round-bottomed, 96-well plates (Costar, Corning, NY) in the presence of 10 μg/mL anti-CD3 mAb and 5 μg/mL anti-CD28 mAb (Biolegend, San Diego, CA) for 72 h. CD4^+^ T cell proliferation was measured by CFSE dilution using FACSCalibur.

### Hypoxic and normoxic G-MDSC culture conditions

A hypoxic environment was created by culturing G-MDSCs with AnaeroGen™ (Thermo Fisher Scientific, Massachusetts, USA) in a sealed box placed in an incubator at 37 °C (O_2_ < 0.1, 5% CO_2_). G-MDSCs cultured directly in the incubator chamber at 37 °C (20% O_2_, 5% CO_2_) were considered normoxia control cells.

### In vivo experiments

Briefly, 1 × 10^6^ G-MDSCs isolated from tumor tissues and subsequently transfected with Pvt1 siRNA or the negative control mixed with 0.8 × 10^6^ LLC cells were implanted via s.c. injection into C57BL/6 mice, and tumor growth was monitored continuously. Tumor volume was calculated using the formula V = 1/2 × a^2^ × b, where ‘a’ represents the smaller diameter and ‘b’ is the larger diameter. The Th1 cell and CTL proportion in spleens, draining lymph nodes (dLNs), and tumor tissues from the tumor-bearing mice were detected via FCM.

### Statistical analysis

The data are expressed as the mean ± SD. The statistical significance of differences between groups was determined via *t* tests and ANOVA using SPSS 19.0 software. Data from all experiments were imported into GraphPad Prism 5.0 (GraphPad, San Diego, CA) to generate bar graphs. Differences were considered to be significant at a *p* level less than 0.05.

## Results

### Pvt1 is highly expressed in tumor-expanded G-MDSCs

**Fig. 1:**
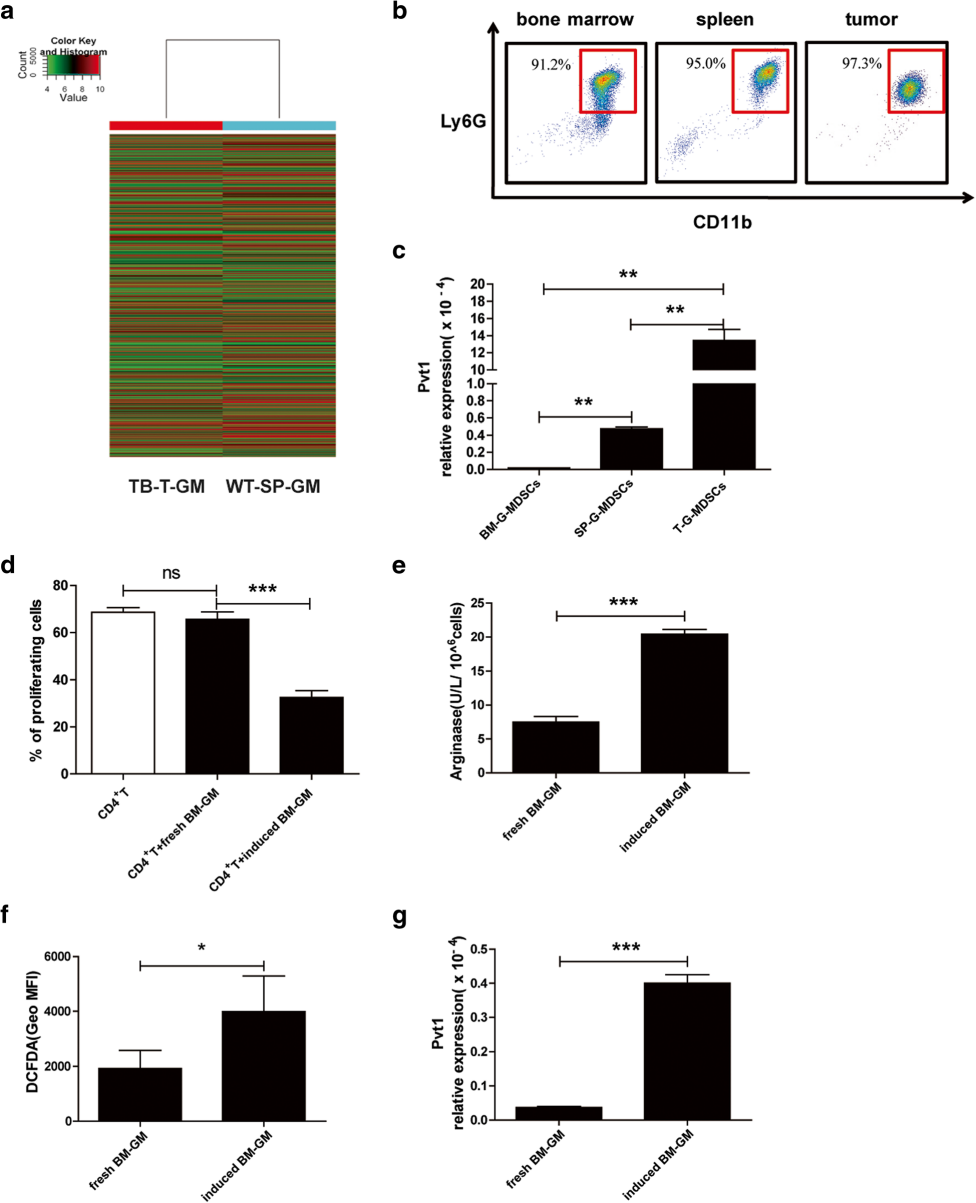
Pvt1 is highly expressed in tumor-expanded G-MDSCs. A total of 2 × 10^^6^ Lewis lung carcinoma cells (LLCs) were introduced via s.c. injection into C57BL/6 mice. After 4 weeks, bone marrow cells, splenocytes and a single-cell suspension derived from tumor tissues were collected, and G-MDSCs were later sorted. Splenocytes from wild-type (WT) C57BL/6 mice were collected, and G-MDSCs were isolated. Hierarchical clustering analysis of lncRNAs and protein-coding RNAs that were differentially expressed (fold change > 2) in G-MDSCs sorted from tumor tissue of Lewis tumor-bearing mice and spleens of WT C57BL/6 mice. **a** Clustering tree for lncRNAs; the expression values are represented in shades of red and green, indicating expression above and below normal values, respectively. **b** The purity of sorted G-MDSCs was determined via flow cytometry by assessing the expression of two surface markers: Ly6G and CD11b. **c** The expression level of Pvt1 in total RNA isolated from G-MDSCs from the bone marrow, spleen and tumor tissues of Lewis-bearing mice was measured by qRT-PCR. Fresh G-MDSCs isolated from bone marrow (BM) from WT C57BL/6 mice served as the control. Bone marrow cells (1 × 10^^6^) from WT C57BL/6 mice were plated in 24-well plates in 1 mL of RPMI 1640 medium containing 10% FBS, 20 ng/mL IL-6 and 20 ng/mL GM-CSF. The cells were then collected, and G-MDSCs were sorted 3 days later. **d** G-MDSCs cocultured with CFSE-labeled CD4^+^ T cells at a ratio of 1:1 in the presence of anti-CD3 mAb and anti-CD28 mAb for 72 h. The proliferation of CD4^+^ T cells was detected by flow cytometry at 488 nm excitation light. **e** Arg1 activity in G-MDSCs induced from BM cells was measured. **f** ROS production in G-MDSCs was analyzed via flow cytometry. **g** The expression level of Pvt1 in G-MDSCs was detected using qRT-PCR. ***p < 0.001, and **p < 0.01; ns: no significance

By comparing expression profile of lncRNAs between G-MDSCs isolated from tumor tissues of Lewis tumor-bearing (TB) mice and spleens from corresponding wild-type (WT) C57BL/6 mice using array-based lncRNA profiling, a large number of lncRNAs were found to be highly expressed in TB mice compared with WT mice. We screened out lncRNA Pvt1, which exhibited one of the 20 largest variations in the microarray (Fig. 1a). In addition, we confirmed the microarray results using qRT-PCR. In accordance with the array data, qRT-PCR analysis showed that Pvt1 expression was approximately 18 times higher in TB mice than in corresponding WT mice. To examine whether upregulation of Pvt1 in Lewis tumor-expanded G-MDSCs could be extrapolated to other tumors, murine CT26 colorectal tumors were established by implanting 1 × 10^^6^ CT26 cells via s.c. injection into BALB/c mice. An increase in the Pvt1 level in G-MDSCs from TB mice relative to that in G-MDSCs from corresponding WT mice was found (Additional file 1: Figure S1*a*).

It is widely known that MDSCs from both spleens and tumor tissues of TB mice effectively suppress the T cell response, but MDSCs from tumor tissues have a significantly stronger suppression effect. Therefore, we sorted G-MDSCs from bone marrow, spleens and tumor tissues of TB mice and analyzed cell purity using flow cytometry. As shown in Fig. 1b, the G-MDSC purity was higher than 90% in all samples, which met the requirements of subsequent experiments. The level of lncRNA Pvt1 in G-MDSCs from tumor tissues was clearly higher than that in G-MDSCs from spleens or bone marrow (Fig. 1c). All these data indicate that lncRNA Pvt1 is highly expressed in G-MDSCs from tumor tissues. To further determine the relationship between the suppressive activity of G-MDSCs and the Pvt1 level in G-MDSCs, we chose G-MDSCs from the bone marrow induced with IL-6 and GM-CSF for further study. Cytokine-induced G-MDSCs had a stronger inhibitory effect on CD4^+^T cell proliferation (Fig. 1d). At the same time, induced G-MDSCs had stronger Arg1 activity and higher ROS levels than G-MDSCs from fresh bone marrow (Fig. 1e and f). The level of Pvt1 was also upregulated in induced G-MDSCs (Fig. 1 g). Thus, the high level of Pvt1 might be related to a stronger immunosuppression effect of G-MDSCs.

As mentioned earlier, MDSCs are divided into two subgroups, of which G-MDSCs are the majority group. We also detected the expression of Pvt1 in G-MDSCs and M-MDSCs and found that the expression level of Pvt1 was not significantly different between G-MDSCs and M-MDSCs from the bone marrow, spleen and tumor tissues (Additional file 1: Figure S2).

### Knockdown of Pvt1 alters the suppressive capacity of G-MDSCs in vitro

**Fig. 2:**
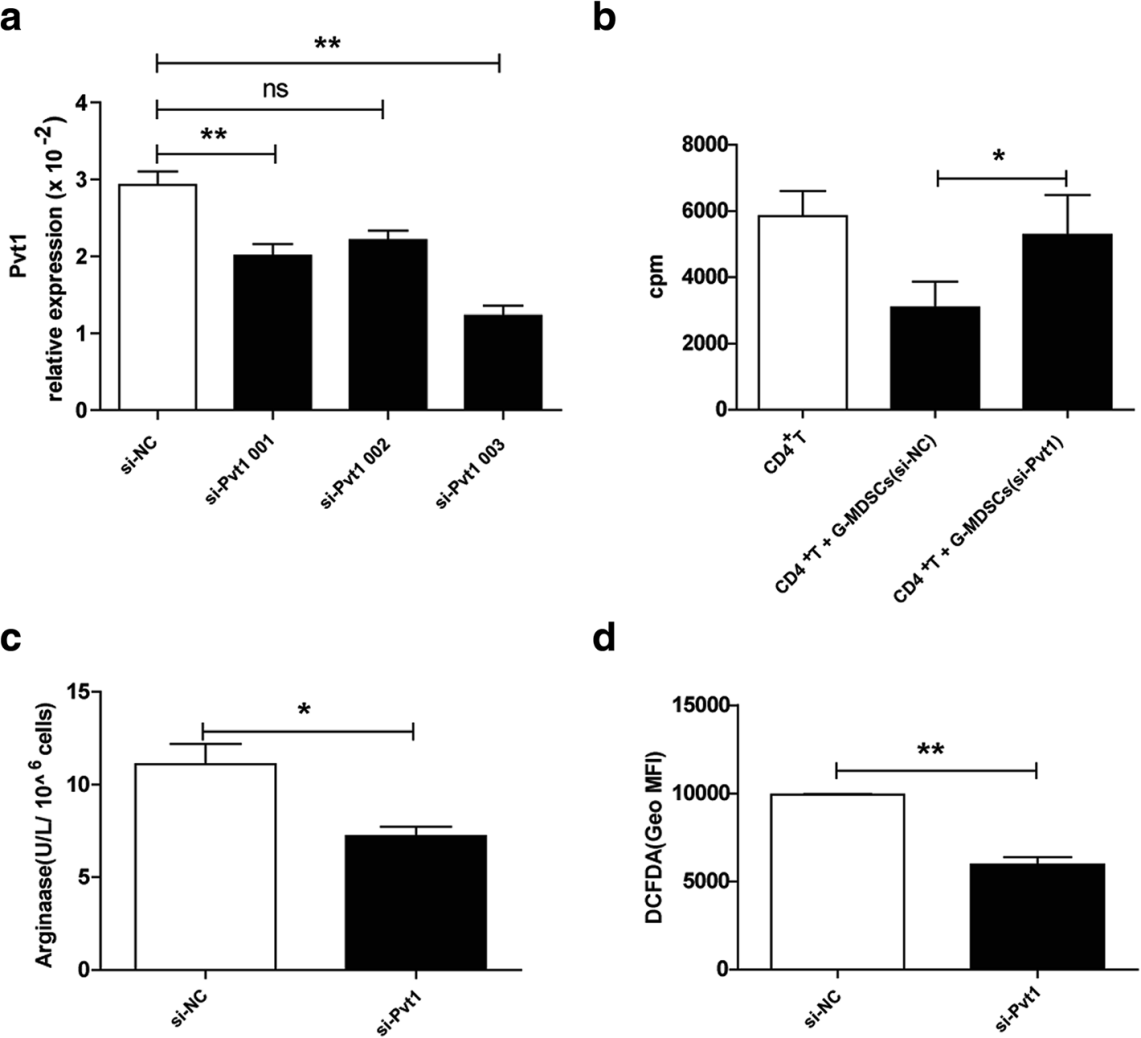
Knockdown of Pvt1 alters the suppressive capacity of G-MDSCs in vitro. G-MDSCs sorted from tumor tissues were obtained from TB mice injected with cells transfected with 50 nM Pvt1 siRNA (si-Pvt1) or negative control (NC) siRNA (si-NC). **a** qRT-PCR confirmed the efficiency of transfection with si-Pvt1. **b** G-MDSCs were transfected with Pvt1 siRNA, and then, the cells were harvested after 6 h and cocultured with CD4^+^T cells at ratio of 1:1 in the presence of anti-CD3 mAb and anti-CD28 mAb for72 h. 3H-thymidine incorporation was used to detect T cells proliferation. **c** Arg1 activity in G-MDSCs transfected with si-Pvt1 was measured. **d** ROS production in G-MDSCs was analyzed via flow cytometry. **p < 0.01, and *p < 0.05; ns: no significance; Geo MFI: geometric mean fluorescent intensity

As described previously, a high level of Pvt1 is associated with stronger immunosuppression induced by G-MDSCs. Therefore, we hypothesized that Pvt1 could regulate the function of G-MDSCs. To investigate the function of Pvt1 in G-MDSCs, G-MDSCs isolated from tumor tissues were transfected with Pvt1-specific small interfering RNA (si-Pvt1) to knockdown Pvt1 expression. Compared with the negative control, the expression of Pvt1 in G-MDSCs transfected with si-Pvt1 was downregulated, especially in cells transfected with si-Pvt1 003 (Fig. 2a). We chose si-Pvt1 003, rather than si-Pvt1 001 or si-Pvt1 002, for use in the following experiments. In addition, we found that G-MDSC-mediated T-cell suppression was weakened after Pvt1 knockdown (Fig. 2b). It has been reported that G-MDSCs suppress T-cell-induced antitumor immune responses through a large number of mechanisms, including through Arg1 and ROS. As shown in Fig. 2c and d, after transfection with si-Pvt1, Arg1 activity and ROS production were clearly decreased in G-MDSCs. Therefore, Pvt1 likely downregulates the immunosuppressive effect of G-MDSCs in vitro.

### C-myc is likely a downstream target of Pvt1 in G-MDSCs

**Fig. 3:**
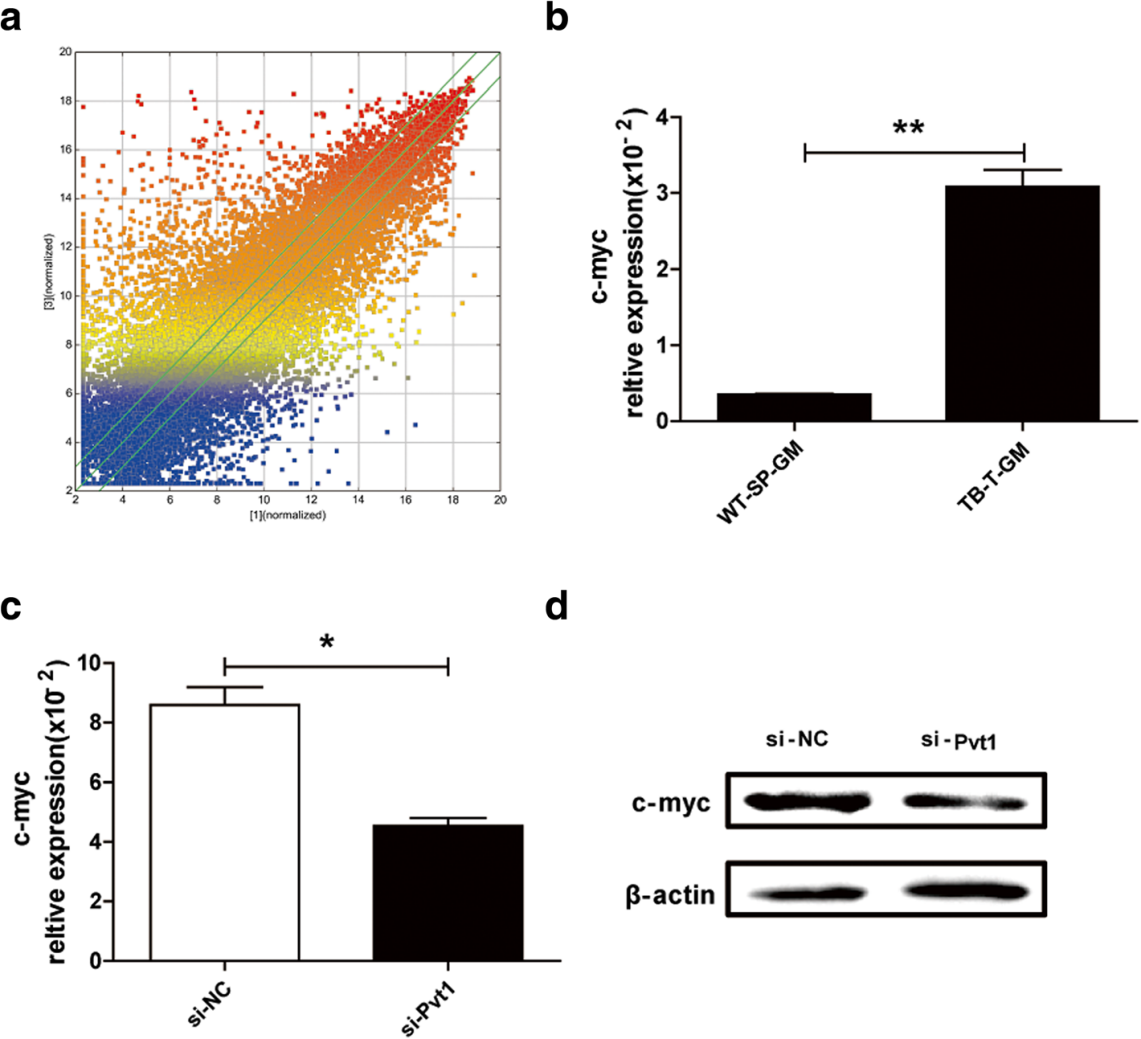
c-myc is a potential downstream target of Pvt1 in G-MDSCs. **a** Scatter plot for protein-coding RNAs. **b** qRT-PCR was used to detect the mRNA level of c-myc in G-MDSCs sorted from spleen of WT mice and tumor tissues of TB mice. **c**,**d** After transfection with si-Pvt1, the mRNA and protein levels of c-myc in G-MDSCs isolated from tumor tissues were measured via qRT-PCR and western blot analyses. **p < 0.01, and *p < 0.05

According to the regulatory mechanisms of lncRNA, intergenic lncRNA can regulate the expression of nearby genes. Pvt1 has been defined as an intergenic lncRNA in many diseases, and c-myc is adjacent to Pvt1 in the chromosome. As predicted by the gene microarray results (Fig. 3a), the mRNA level of c-myc was distinctly higher in G-MDSCs isolated from tumor tissues of TB mice than in G-MDSCs isolated from spleens of WT mice (Fig. 3b). In murine CT26 colorectal tumors, the same results were observed (Additional file 1: Figure S1*b*). In keeping with the Pvt1 findings, the c-myc level in G-MDSCs from tumor tissues was considerably higher than in the spleens and bone marrow from TB mice (Additional file 1: Figure S3*a*). Additionally, c-myc levels were increased in G-MDSCs induced from bone marrow cells with IL-6 and GM-CSF compared with G-MDSCs from fresh bone marrow (Additional file 1: Figure S3*b*). All these data suggested that the alteration in c-myc expression was in accordance with the change in Pvt1 levels; thus, c-myc might be a downstream target of Pvt1 in G-MDSCs. For further certification, we knocked down Pvt1 in G-MDSCs with si-Pvt1 and then measured the mRNA and protein levels of c-myc. As shown in Fig. 3c and d, both the mRNA and protein levels of c-myc were decreased in G-MDSCs transfected with si-Pvt1. The results indicate that c-myc is potentially a downstream target of Pvt1 in G-MDSCs, but whether c-myc participates in regulation of G-MDSC function through Pvt1 requires further study.

### Pvt1 knockdown reduces the ability of G-MDSCs to accelerate tumor progression and inhibit antitumor immune responses

**Fig. 4:**
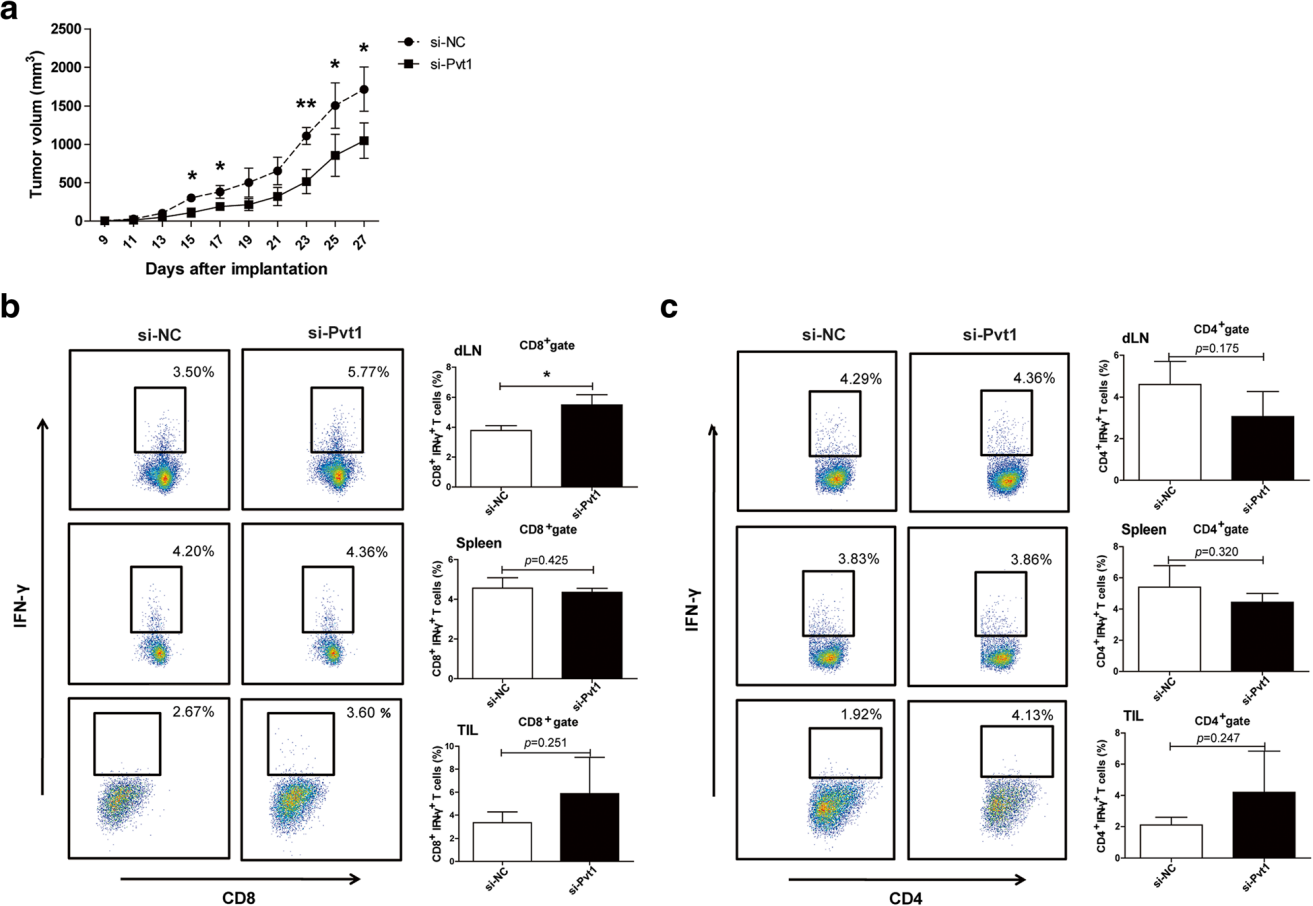
Pvt1 knockdown reduces the ability of G-MDSCs to accelerate tumor progression and inhibit antitumor immune responses. Two groups of mice were given a s.c. injection of a mixture of LLCs and G-MDSCs transfected with si-Pvt1 (si-Pvt1 group) or si-NC (si-NC group). **a** Tumor volume was measured at the indicated time. **b**, **c** The proportions of CD8^+^IFN-γ^+^ CTLs and CD4^+^IFN-γ^+^ Th1 cells from draining lymph nodes, spleens and tumor tissues were analyzed via flow cytometry. **p < 0.01, and *p < 0.05

G-MDSCs isolated from tumor tissues of tumor-bearing C57BL/6 mice were transfected with si-Pvt1 in vitro. Then, 1 × 10^^6^ G-MDSCs transfected with si-Pvt1 mixed with 0.8 × 10^^6^ Lewis lung carcinoma (LLC) cells were implanted via s.c. injection into WT C57BL/6 mice. As shown in Fig. 4a, tumor growth was significantly reduced in mice injected with G-MDSCs transfected with si-Pvt1 (si-Pvt1 group) compared with mice injected with G-MDSCs transfected with the negative control (si-NC group). In the tumor microenvironment, MDSCs primarily suppress the antitumor response by inhibiting T cell responses. We investigated whether alteration of Pvt1 expression in G-MDSCs could regulate CTL and Th1 responses in vivo. Therefore, the proportions of CD8^+^ IFN-γ^+^ CTLs and CD4^+^ IFN-γ^+^ Th1 cells in draining lymph nodes (dLNs), spleens and tumor tissues were detected by flow cytometry. We found that the proportion of CTLs in dLNs from the si-Pvt1 group was much higher than that in dLNs from the si-NC group, and the proportions of CTLs and Th1 cells in tumor tissues exhibited a moderate increase in the si-Pvt1 group compared with the si-NC group (Fig. 4b and c).

### HIF-1α upregulates Pvt1 expression in G-MDSCs under hypoxic stress

**Fig. 5:**
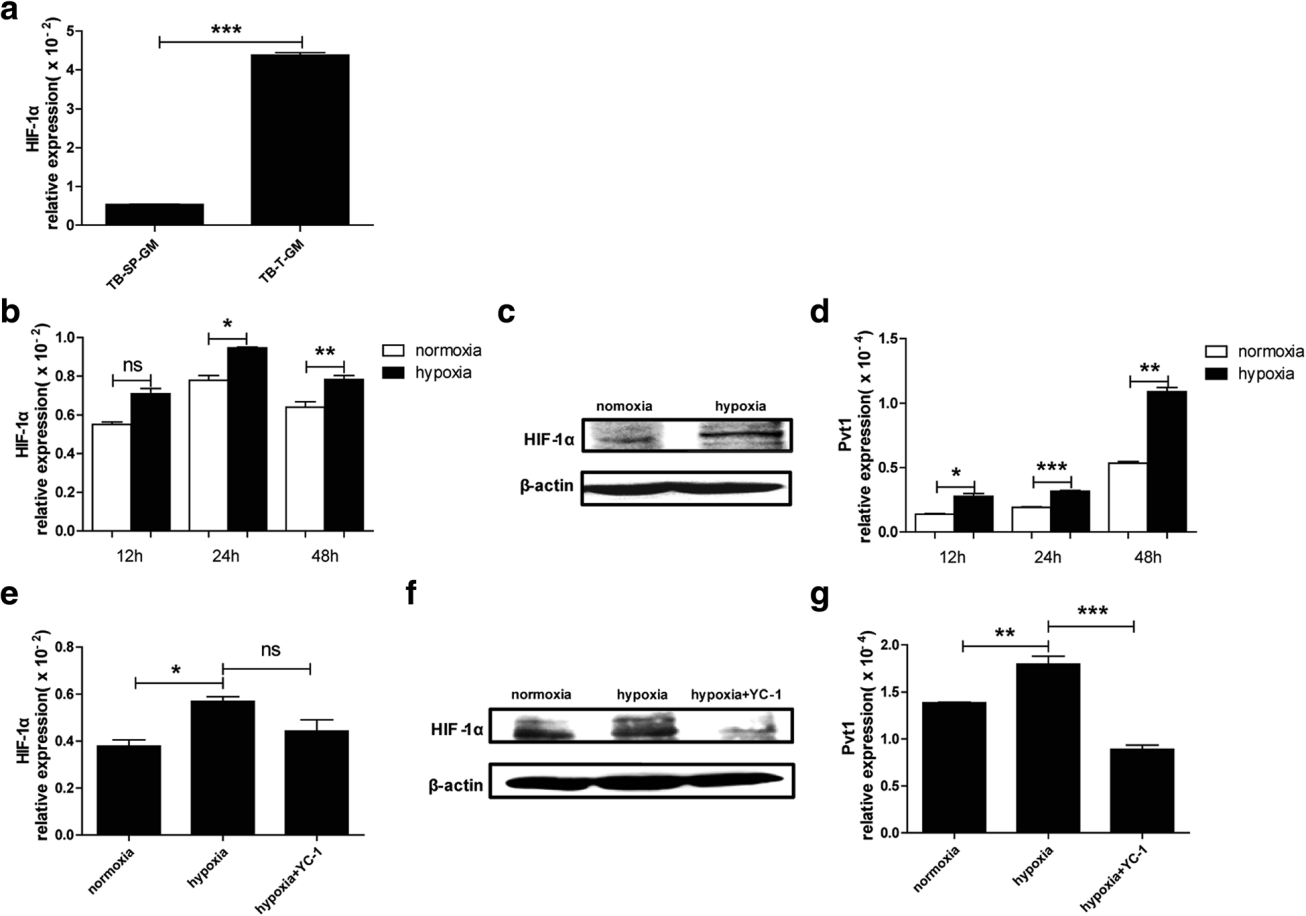
HIF-1α upregulates Pvt1 expression in G-MDSCs under hypoxic stress. **a** The mRNA level of HIF-1α in G-MDSCs sorted from spleens and tumor tissues of TB mice was detected using qRT-PCR. G-MDSCs isolated from spleens of TB mice were cultured in an incubator at 37 °C (20% O_2_, 5% CO_2_) (normoxic conditions) or in a sealed box containing an anaerobic bag to consume oxygen (O_2_ < 0.1%, 5% CO_2_) (hypoxic conditions). **b, c** The mRNA and protein levels of HIF-1α were measured via qRT-PCR and western blot (WB) analyses, respectively. **d** Pvt1 expression was analyzed via qRT-PCR. YC-1, a specific inhibitor of HIF-1α, was used to block hypoxia. **e, f** HIF-1α and (**g**) Pvt1 expression in the normoxia, hypoxia, and hypoxia+YC-1 groups were detected via qRT-PCR and WB analyses. ***p < 0.001, **p < 0.01, and *p < 0.05; ns: no significance

As mentioned above, the expression of Pvt1 was higher in tumor-infiltrating G-MDSCs than in splenic G-MDSCs. We explored whether the tumor microenvironment might regulate the expression of Pvt1 in G-MDSCs, and which factor likely regulates the Pvt1 level. Considering the hypoxic conditions in the tumor microenvironment and that the primary molecular response to hypoxia is elicited through HIF-1α, we examined HIF-1α expression and found that it was higher in tumor-infiltrating G-MDSCs than in splenic G-MDSCs (Fig. 5a). We exposed splenic G-MDSCs to hypoxic conditions in vitro and measured HIF-1α expression to determine the success of the hypoxia treatment. Both the mRNA and protein levels of HIF-1α were upregulated in G-MDSCs under hypoxic conditions (Fig. 5b and c). Meanwhile, G-MDSCs exposed to hypoxic conditions expressed higher levels of Pvt1 than G-MDSCs under to normoxic conditions (Fig. 5d), which confirmed our hypothesis. In addition, consistent with the Pvt1 levels, the expression of c-myc in G-MDSCs was also upregulated under hypoxia (Additional file 1: Figure S4*a*). To adequately demonstrate whether HIF-1α regulates Pvt1 expression, we used a specific inhibitor of HIF-1α, YC-1, to block upregulation of HIF-1α under hypoxic conditions. When G-MDSCs were exposed to hypoxic conditions, we added YC-1 to inhibit HIF-1α expression. The results showed that HIF-1α expression was decreased (Fig. 5e and f). In addition, the upregulation of Pvt1 and c-myc under hypoxia was restored by treatment with the HIF-1α inhibitor (Fig. 5g and Additional file 1: Figure S4*b*). Taken together, these results suggest that the expression of Pvt1 in G-MDSCs might be regulated by HIF-1α under hypoxic stress.

## Discussion

Hypoxia, or a low level of oxygen, is one of the hallmarks of the tumor microenvironment. Hypoxia-inducible factors (HIFs) play a critical role in regulation of cellular responses to hypoxia. In recent years, it has been reported that HIF-1α plays a prominent role in immune cells and regulates immune responses in the tumor microenvironment. A large number of immunosuppressive cells, including MDSCs, tumor-associated macrophages (TAMs), and regulatory T cells (Tregs), are recruited to hypoxic zones in solid tumors [35]. In addition, the differentiation and function of immune effector cells, such as dendritic cells (DCs) and tumor infiltrating lymphocytes (TILs), can be changed through modulation of the expression of costimulatory receptors and the type of cytokines produced by these cells, which is beneficial for tumor progression [36].

The role of hypoxia in MDSC regulation has been reported previously. David Kung et al. [37] found that HIF upregulate chemokine (C-C motif) ligand 26 (CCL26) in cancer cells to recruit chemokine (C-X3-C motif) receptor 1 (CXC3R1)-expressing MDSCs to the primary tumor in hepatocellular carcinoma. Tumor-infiltrating MDSCs are more immunosuppressive than splenic MDSCs, primarily due to an increase in Arg1 activity and nitric oxide production via HIF-1α in these cells [38]. In this study, we found for the first time that Pvt1 is a target of HIF-1α under hypoxia in G-MDSCs from Lewis lung carcinoma mice. Pvt1 was clearly upregulated under hypoxic conditions by HIF-1α, and inhibition of HIF-1α by YC-1 apparently reduced Pvt1 expression in G-MDSCs. Thus, Pvt1 might be a target of HIF-1α, which regulates antitumor immune responses by modulating the function of G-MDSCs.

The plasmacytoma variant translocation 1 (Pvt1) gene was initially identified as a transcriptional unit encoded by a sequence homologous to the PVT1 gene, and Pvt1 promoted cell proliferation and cell cycle progression and inhibited apoptosis when overexpressed and amplified in a variety of cancers, indicating that it is an oncogene [26, 39, 40]. In patients with various types of cancer, a higher PVT1 level indicates a significantly poorer overall survival time, and PVT1 expression is a novel biomarker for cancer diagnosis and prognosis [41, 42]. MDSCs are among the primary immunosuppressive cells in cancer, and various therapeutic strategies targeting MDSCs are now being explored, including inhibition of MDSC expansion, direct elimination of MDSCs, promotion of MDSC differentiation, and inhibition of the immunosuppressive capacity of MDSCs. However, the regulatory effect of Pvt1 on MDSC function has not been reported to date.

To shed light on the potential effect of Pvt1 on G-MDSC activity, we used a murine Lewis lung carcinoma model to investigate the detailed mechanism by which Pvt1 affects G-MDSCs. We found that the expression of Pvt1 was higher in G-MDSCs isolated from tumor tissues of Lewis-bearing mice than in spleens from wild-type mice. Previous studies have demonstrated that G-MDSCs from the tumor site have significantly stronger immune suppression activity than those from spleens [38]. We speculated that the level of Pvt1 might be associated with the suppressive capacity of G-MDSCs. G-MDSCs induced from bone marrow cells by treatment with IL-6 and GM-CSF exhibited stronger immunosuppressive capacity and higher Pvt1 expression than fresh G-MDSCs sorted from bone marrow, which exhibited no immunosuppression activity. These data indicate that the level of Pvt1 might be related to the degree of immunosuppression induced by G-MDSCs. To further define the role of Pvt1 in the function of G-MDSCs, Pvt1-specific siRNA was used to inhibit Pvt1 expression. We found that G-MDSC-mediated T cell suppression was altered after Pvt1 knockdown. Many researchers have noted that G-MDSCs suppress T-cell-induced antitumor immune responses primarily through Arg1 and ROS. As expected, after transfection with si-Pvt1, both the activity of Arg1 and the production of ROS decreased in G-MDSCs. As mentioned above, Pvt1 likely promotes tumor progression by promoting proliferation and inhibiting apoptosis in most tumor cells. However, whether Pvt1 regulates cell cycle progression and apoptosis in G-MDSCs requires further research.

It has been reported that Pvt1 has three main molecular mechanisms of action: encoding microRNAs, taking part in DNA rearrangements, and interacting with MYC [26, 43]. Pvt1 can act as a fusion partner, interfering with the regulation of a large number of oncogenes through DNA rearrangements to promote tumorigenesis. The Pvt1 locus has recently been found to contain a cluster of more than six microRNAs, namely, miR-1204, miR-1205, miR-1206, miR-1207-5p, miR-1207-3p, and miR-1208. These microRNAs participate in tumor progression [44, 45]. Coamplification of Pvt1 and MYC has been found in abundant human and animal tumors over the past few decades [46, 47]. It is widely known that the proto-oncogene, c-myc, plays a key role in cell proliferation, apoptosis, and terminal differentiation of hematopoietic cells via regulating transcription of downstream target genes [48]. In recent years, c-myc has been reported to play a critical role in alternative activation of human macrophages and is proposed as one of the M2 macrophage markers [49, 50]. Pyzer AR and colleagues reported that c-myc can promote MDSC proliferation by upregulating cyclin D2 and E1 in acute myeloid leukemia (AML) [51]. Considered the above data, we wanted to confirm whether c-myc was a target of Pvt1 in regulation of G-MDSCs. In our study, we found coamplification of Pvt1 and c-myc in LLC tumor-bearing mice. Knockdown of Pvt1 in G-MDSCs reduced both the mRNA and protein levels of c-myc, indicating that c-myc is likely a downstream target of Pvt1 in G-MDSCs, but, whether c-myc participates in regulation of G-MDSC function or differentiation through Pvt1 needs to be further confirmed.

## Conclusions

In this study, we report for the first time that HIF-1α upregulates Pvt1 expression in G-MDSCs under hypoxia. Pvt1 plays a critical role in regulation of the immunosuppressive capacity of G-MDSCs. Pvt1 knockdown decreased the level of Arg1 and ROS in G-MDSCs and restored antitumor T-cell responses. In conclusion, our results indicate that targeting of Pvt1 could weaken G-MDSC-mediated immunosuppression, which might be further validated as a potential therapeutic strategy.

## Additional file

Additional file 1: **Figure S1.** Pvt1 and c-myc are highly expressed in CT26 tumor-expanded G-MDSCs**. Figure S2.** The expression of Pvt1 in G-MDSCs and M-MDSCs was not significantly different. **Figure S3.** c-myc is highly expressed in G-MDSCs with stronger suppression. **Figure S4.** c-myc expression changes are consistent with changes in Pvt1 expression in G-MDSCs under hypoxic stress. (PDF 264 kb)

## Abbreviations

Arg1: Arginase 1
FCM: flow cytometry
GM-CSF: Granulocyte-macrophage colony-stimulating factor
G-MDSCs: granulocytic MDSCs
HIF-1α: Hypoxia-inducible factor (HIF)-1α
IL-6: Interleukin-6
LncRNAs: Long non-coding RNAs
MDSCs: myeloid-derived suppressor cells
M-MDSCs: monocytic MDSCs
Pvt1: plasmacytoma variant translocation
ROS: reactive oxygen species
