# Supplementary material for: Long noncoding RNA Pvt1 regulates the immunosuppression activity of granulocytic myeloid-derived suppressor cells in tumor-bearing mice
Source: Mol Cancer. 2019 Mar 30;18:61. doi: 10.1186/s12943-019-0978-2 (PMC6441229; doi:10.1186/s12943-019-0978-2)
Supplement: Supplementary file 1 — Figure S1. Pvt1 and c-myc are highly expressed in CT26 tumor-expanded G-MDSCs. Figure S2. The expression of Pvt1 in G-MDSCs and M-MDSCs was not significantly different. Figure S3. c-myc is highly expressed in G-MDSCs with stronger suppression. Figure S4. c-myc expression changes are consistent with changes in Pvt1 expression in G-MDSCs under hypoxic stress. (PDF 264 kb) [file 12943_2019_978_MOESM1_ESM.pdf]

Supplementary Figures

Supplementary Figure 1

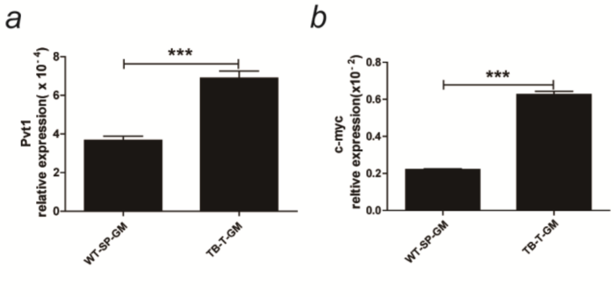

Supplementary Figure 1. Pvt1 and c-myc are highly expressed in CT26 tumor-expanded G-MDSCs.

In total, 2×10^6 CT26 colon cancer cells were implanted via *s.c.* injection into BALB/c mice. After 4 weeks, a single-cell suspension derived from tumor tissues was obtained, and G-MDSCs were sorted. Splenocytes from wild-type (WT) BALB/c mice were collected, and G-MDSCs were isolated. (***a***) Pvt1 and (***b***) c-myc expression in total RNA was measured via qRT-PCR. ***$p < 0.001$.

Supplementary Figure 2

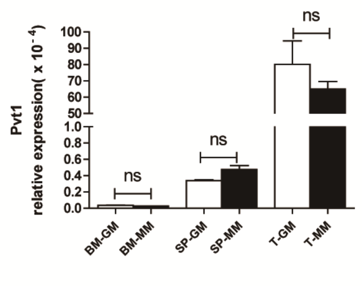

Supplementary Figure 2. The expression of Pvt1 in G-MDSCs and M-MDSCs was not significantly different.

In total, 2×10^6 Lewis lung carcinoma (LLC) cells were implanted via *s.c.* injection into C57BL/6 mice. After 4 weeks, bone marrow cells, splenocytes and single-cell suspension of tumor tissues were collected. Then, G-MDSCs and M-MDSCs were isolated using an MDSC Isolation Kit. The expression level of Pvt1 in total RNA was detected using qRT-PCR. ns: no significance.

Supplementary Figure 3

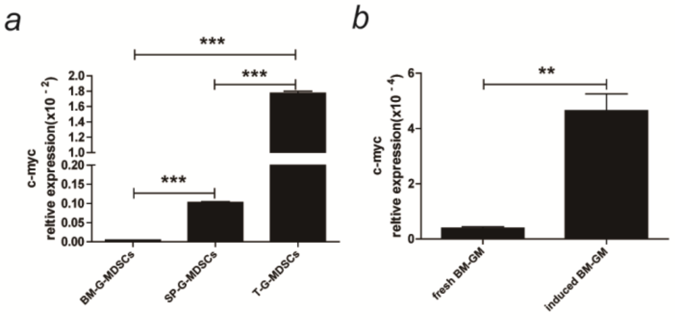

Supplementary Figure 3. c-myc is highly expressed in G-MDSCs with stronger suppression.

In total, $2\times10^{\wedge 6}$ Lewis lung carcinoma (LLC) cells were implanted via *s.c.* injection into C57BL/6 mice. After 4 weeks, bone marrow cells, splenocytes and a single-cell suspension of tumor tissues were obtained, and G-MDSCs were isolated. (***a***) qRT-PCR was used to detect the mRNA level of c-myc. Fresh G-MDSCs isolated from bone marrow (BM) from WT C57BL/6 mice served as the control. Bone marrow cells ($1\times10^{\wedge 6}$) from WT C57BL/6 mice were plated in 24-well plates in 1 ml of RPMI 1640 medium with 10% FBS, 20 ng/ml IL-6 and 20 ng/ml GM-CSF, and after 3 days, the cells were collected, and G-MDSCs were sorted. (***b***) The mRNA level of c-myc in total RNA was measured via qRT-PCR. ***$p < 0.001$, and **$p < 0.01$.

Supplementary Figure 4

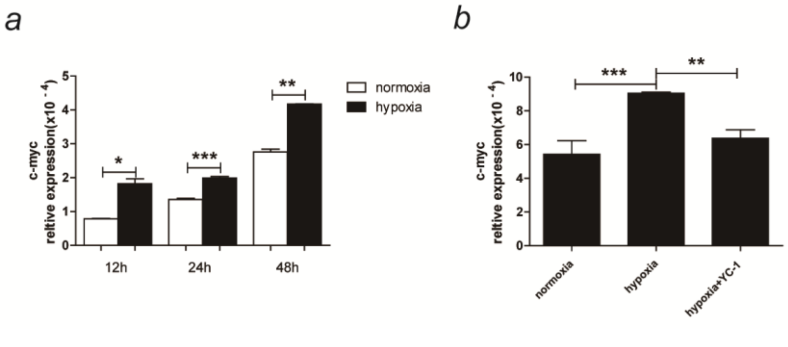

Supplementary Figure 4. c-myc expression changes are consistent with changes in Pvt1 expression in G-MDSCs under hypoxic stress.

G-MDSCs isolated from spleens of TB mice were cultured in an incubator at 37 ℃ (20% O_2, 5% CO_2) (normoxic conditions) or in a sealed box containing an anaerobic bag to consume oxygen (O_2 < 0.1%, 5% CO_2) (hypoxic conditions). (***a***) The c-myc mRNA level was measured via qRT-PCR. YC-1, a specific inhibitor of HIF-1α, was used to block hypoxia. (***b***) The mRNA level of c-myc was detected via qRT-PCR in G-MDSCs in the normoxia, hypoxia, and hypoxia+YC-1

groups. ***$p < 0.001$, **$p < 0.01$, and *$p < 0.05$.
